# Association Study on *ADAM33* Polymorphisms in Mite-Sensitized Persistent Allergic Rhinitis in a Chinese Population

**DOI:** 10.1371/journal.pone.0095033

**Published:** 2014-04-21

**Authors:** Ruo-Xi Chen, Wen-Min Lu, Lu-Ping Zhu, Mei-Ping Lu, Mei-Lin Wang, Yun-Li Wang, Xin-Yuan Chen, Xin-Jie Zhu, Min Yin, Zheng-Dong Zhang, Lei Cheng

**Affiliations:** 1 Department of Otorhinolaryngology, The First Affiliated Hospital, Nanjing Medical University, Nanjing, China; 2 Department of Molecular and Genetic Toxicology, School of Public Health, Nanjing Medical University, Nanjing, China; 3 International Centre for Allergy Research, Nanjing Medical University, Nanjing, China; Tor Vergata University of Rome, Italy

## Abstract

**Background:**

The *ADAM33* gene has been identified as a potentially important asthma candidate gene and polymorphisms in this gene have been shown to be associated with asthma and seasonal allergic rhinitis.

**Objective:**

To assess whether the *ADAM33* polymorphisms are associated with persistent allergic rhinitis (PER) due to house dust mites in a Chinese population.

**Methods:**

In a hospital-based case-control study of 515 patients with mite-sensitized PER and 495 healthy controls, we genotyped seven single nucleotide polymorphisms (SNPs) in *ADAM33*. Serum levels of eosinophil cationic protein, total IgE and allergen-specific IgE against *Dermatophagoides pteronyssinus* and *Dermatophagoides farinae* were measured by the ImmunoCAP assays.

**Results:**

In the single-locus analysis, three polymorphisms, rs3918392 (F1), rs528557 (S2) and rs2787093, were significantly associated with mite-sensitized PER. SNP S2 was associated with significantly increased risk both of asthmatic and nonasthmatic mite-sensitized PER. In the combined genotypes analysis, individuals with 2–4 risk alleles had a significantly higher risk of mite-sensitized PER (adjusted OR = 1.99, 95% CI = 1.50–2.62) than those with 0–1 risk alleles. Haplotype-based association analysis revealed that the ACAGCCT haplotype might have potential to protect against mite-sensitized PER (adjusted OR = 0.67; 95% CI = 0.49–0.90).

**Conclusions:**

Polymorphisms in the *ADAM33* gene may contribute to susceptibility of mite-sensitized PER in this Chinese population.

## Introduction

Allergic rhinitis (AR) is defined as a nasal disorder induced by immunoglobulin E (IgE)-mediated immune response and characterized by nasal symptoms including rhinorrhoea, sneezing, nasal obstruction, and itching [1]. It is one of the most common atopic diseases throughout the world. In China, the prevalence of AR ranges from 8.7% to 24.1% [2]. Meanwhile, epidemiologic studies have consistently shown that rhinitis and asthma often coexist in one patient [3], [4], [5]. AR and asthma, respectively affecting upper and lower airway, where mucosa present similarities and complement in function, both manifest atopy symptoms and likely share a common genetic background [6], suggesting the concept of ‘one airway, one disease’ [7].

AR and asthma are both multi-factorial diseases with genetic as well as environmental factors influencing disease progression. A gene encoding a disintegrin and metalloprotease (ADAM) 33 located on chromosome 20p13 was identified by positional cloning as a putative candidate gene for the development of asthma and bronchial hyperresponsiveness (BHR) [8]. Selective expression of ADAM33 in lung mesenchymocytes suggests that alterations in its activity may underlie abnormalities in the function of airway smooth muscle cells and fibroblasts linked to BHR and airway remolding [9]. One possible explain is that ADAM33 may serve as a cell surface ‘sheddase’ to release growth factors such as transforming growth factor (TGF)-β and modify cell-surface receptor expression to induce the proliferation of airway mesenchymocyte [10].

Significant associations between single nucleotide polymorphisms (SNPs) of the *ADAM33* gene and asthma in ethnically diverse populations have been reported [8], [11], [12], [13], [14], [15], [16], [17], [18], [19]; however, little attention was given to rhinitis. Six SNPs (F+1, L−1, S2, T1, T2 and T+1) in *ADAM33* were reported to have significant association in seasonal allergic rhinitis (SAR) induced by Japanese cedar pollen [20], while three SNPs (T1, T+1 and V4) were reported to be associated with AR or concomitant AR and Asthma in a Chinese population [12], [21]. Recently, a study showed that haplotypes of ADAM33 polymorphisms exhibited significant association with AR among Jordanians [22].

To confirm the association between *ADAM33* polymorphisms and AR induced by house dust mites (HDM), and to examine the association with clinical phenotypes including rhinitis severity, concomitant asthma, and serum total IgE levels, we genotyped and analyzed seven SNPs of the *ADAM33* gene in a Chinese population.

## Materials and Methods

### Subjects

The 515 unrelated individuals (342 males and 173 females) with persistent allergic rhinitis (PER) were aged from 3 to 66 years with a mean age of 19.68±12.6 years. The 495 unrelated healthy controls (299 males and 196 females) were aged from 3 to 65 years with a mean age of 20.10±12.61 years. All the cases and controls were recruited from Jiangsu and Anhui provinces, eastern China. All patients were enrolled in the First Affiliated Hospital of Nanjing Medical University (Nanjing, China) from May 2008 to January 2013. PER was diagnosed according to the guidelines of Allergic Rhinitis and its Impact on Asthma (ARIA) 2008 update [1]. A questionnaire was used to obtain information of disease condition and status, including history, family history, symptoms and concomitant diseases from each PER case. In this study, control subjects were recruited from the hospital who seeking health care or doing routine health examinations, and were frequency matched with the cases by age (±5 years) and sex. Individuals selected for controls must meet all the following criteria: (i) no symptoms or history of rhinitis or other nasal diseases; (ii) no symptoms or history of asthma or other atopic disorders; (iii) have negative allergen-specific IgE in serum (Phadiatop<0.35 kU_A_/L); (iv) absence of first-degree relatives with a history of AR or other allergic diseases. Subjects of all only Han Chinese were included in this study and the participation rate was approximately 95%. The research protocol was approved by the ethical review board of Nanjing Medical University, and written informed consent was obtained from all participants.

### Clinical Parameters

A visual analogue scales (VAS) ranging from 0 (not at all bothersome) to 10 cm (extremely bothersome) was used to assess the severity of total nasal symptoms (VAS score) as well as individual symptoms including rhinorrhoea, sneezing, nasal obstruction, and nose and eye itching. Patients with VAS score ≤5 were diagnosed as mild rhinitis and those with VAS score >5 as moderate/severe [23].

About 5 mL venous blood samples were obtained from each participant for *in vitro* allergy testing. The serum total IgE, specific IgE, and eosinophil cationic protein (ECP) levels were measured with ImmunoCAP100 assays (Phadia, Uppsala, Sweden), according to the manufacturer’s standard procedures. Total IgE and ECP were determined in all subjects. Specific IgE antibodies to common inhalant allergens including *Dermatophagoides pteronyssinus* (*Der p*; d1), *Dermatophagoides farinae* (*Der f*; d2), cat dander (e1), dog dander (e5), *Blatella germanica* (i6), *Alternaria alternata* (m6), *Ambrosia elatior* (w1), and *Artemisia vulgaris* (w6) were determined in patients with PER.

### SNP Selection and Genotyping

Seven SNPs in the *ADAM33* gene, including rs3918392 (F1), rs528557 (S2), rs2280091 (T1), rs2280090 (T2), rs3918400 (V2) rs2787094 (V4), and rs2787093 (located in 3′ near gene), were selected by using genotype data obtained from unrelated Han Chinese in Beijing (CHB) individuals in the HapMap (HapMap Data Rel 28 Phase II+III, August 10, on NCBI B36 assembly, dbSNP b126) and based on previously published studies [8], [12], [20], [21] identifying putative risk alleles. Genotyping was performed with the TaqMan SNP Genotyping Assay using the 384-well ABI 7900HT Real-Time PCR System (Applied Biosystems, Foster City, CA, USA). More than 15% of the samples were randomly selected for confirmation, and the results were 100% concordant. The primers and TaqMan probes are shown in Table 1.

**Table 1:** Primers and probes for genotypes screening by TaqMan allelic discrimination.

NCBI rs No.	Base change	Primers*	Probes*
rs3918392	A>G	F: CTCAACCCACGAGATCTTTCG	G allele: FAM-CCTGGAAAGGAGCCT-MGB
		R: GGTCATGCCCGCTTTGTT	A allele: HEX-CCTGGAAAGGAACC-MGB
rs528557	C>G	F: CTTCCTGCTGGCCATGCT	G allele: FAM-TGCTCCCAGGGGC-MGB
		R: CCTGGGAGTCGGTAGCAACA	C allele: HEX-TGCTCCCAGGCGC-MGB
rs2280091	A>G	F: CCCAAAGATGGCCCACACA	C allele: FAM-CACCCCACGGAGTT-MGB
		R: GGCATGAGCCCTTCCCTTCT	T allele: HEX- ACCCCATGGAGTTGG-MGB
rs2280090	A>G	F: CCCAAAGATGGCCCACACA	C allele: FAM-TGGACAGCCCTGGC-MGB
		R: GGCATGAGCCCTTCCCTTCT	T allele: HEX-TGGACAGTCCTGGC-MGB
rs3918400	C>T	F: GCAGGCAGCTTGGAAGTTTC	T allele: FAM-AGTGGAGCTTTGACC-MGB
		R: TCTAATGTGGCTCTGGGTTCCT	C allele: HEX-TGGAGCTTCGACCC-MGB
rs2787094	C>G	F: TGGCCAGAAGCTAGTGGTCCT	C allele: FAM-CTCCCCTGCAGCCT-MGB
		R: CAGGAAGGAAGGTCCCCAAA	G allele: HEX-CTCCCCTGGAGCCT-MGB
rs2787093	C>T	F: GTGGGGTGGTGGCTGATG	A allele: FAM-AGCCTAGATGGCAGC-MGB
		R: AAAGAGGGTGTTCAAACTAGGCAA	G allele: HEX-CCGAGCCTAGGTGG-MGB

*The alleles were arrayed as the location of the primers or probes from 5′to 3′.

### Statistical Analysis

Differences in the distributions of demographic characteristics, selected variables, and frequencies of *ADAM33* genotypes between the cases and controls were evaluated using Student’s *t*-test (for continuous variables) or the Chi-square test (for categorical variables). Serum levels of total IgE and ECP were transformed into logarithmic model for normalize the distribution. Percentiles were calculated based on all available subjects with existing total IgE measurements (n = 973) and logarithmic total IgE 90th percentile (999.9 kU/L) was used as dependent variable (above/below the 90th percentile) with two categories denoted as ‘high’ and ‘low’ total IgE levels, respectively [24]. Hardy-Weinberg equilibrium (HWE) of the genotype distribution among control groups was tested by a goodness-of-fit Chi-square test. Chi-square test and one-way ANOVA were used to assess the difference between the *ADAM33* polymorphisms and clinical phenotypes. The crude and adjusted odds ratios (ORs) and 95% confidence intervals (CIs) were obtained to assess the association between the *ADAM33* polymorphisms and risk of mite-sensitized PER using unconditional univariate and multivariate logistic regression models. The multivariate adjustment included age and sex. The combined genotypes were further stratified by subgroups of age, sex, asthma, history, VAS score, total IgE and HDM-specific IgE levels. The computation of linkage disequilibrium (LD) between SNPs was estimated using the normalized measure of allelic association *D*′ and *r* ^2^, and the characterization of these patterns was determined using Haploview 4.0 software [25]. EM algorithm (SAS 9.1.3 PROC HAPLOTYPE) was used to infer haplotype frequencies based on the observed *ADAM33* genotypes. All of the statistical analyses were carried out with SAS software version 9.1.3 (SAS Institute, Cary, NC, USA), unless indicated otherwise. *P*-value <0.05 was considered statistically significant.

## Results

### Characteristics of the Study Subjects

The frequency distributions of selected characteristics of the PER cases and healthy controls are presented in Table 2. The cases and controls appeared to be well matched on age (*P* = 0.579) and sex (*P = *0.086). Apparently, serum levels of ECP (14.3 [5.6–30.1] µg/L) and total IgE (256.0 [120.2–573.0] kU/L) in cases were significantly higher (*P*<0.001) than those in controls (4.8 [3.1–7.5] µg/L and 26.8 [11.1–52.2] kU/L, respectively). In patients with PER, the serum level of allergen-specific IgE against *Der p* and *Der f* was 27.4 (5.0–69.4) kU_A_/L and 23.1 (5.0–63.3) kU_A_/L, respectively. Of the patients, 277 (53.8%) cases were mild and 270 (52.4%) were moderate/severe according to VAS score, and 130 (30.1%) cases reported to have concomitant asthma and 306 (70.2%) cases had family history of allergic diseases. These variables were further adjusted in the multivariate logistic regression analysis for any residual confounding effect.

**Table 2:** Distribution of selected variables among cases and controls.

Variables	Cases (*n* = 515)		Controls (*n* = 495)		*P*
	N	%	N	%	
Age (years), mean ± SD	19.68±12.6		20.10±12.61		0.597*
Sex					
Male	342	66.4	299	60.4	0.086*
Female	173	33.6	196	39.6	
Serum total IgE (kU/L), median (IQR)	265.0 (120.2–573.0)		26.8 (11.1–52.2)		<0.001†
Eosinophil cationic protein (µg/L), median (IQR)	14.3 (5.6–30.1)		4.8 (3.1–7.5)		<0.001†
Duration of rhinitis (years), mean ± SD	6.62±5.87				
Total nasal symptoms (VAS score), mean ± SD	5.23±2.41				
Concomitant asthma‡					
Yes	130	30.1			
No	304	70.0			
Family history of allergic diseases‡					
Yes	306	70.2			
No	130	29.8			
Specific IgE (kU_A_/L), median (IQR)					
* Dermatophagoides pteronyssinus*	27.4 (5.0–69.4)				
* Dermatophagoides farinae*	23.1 (5.0–63.3)				

*Derived from two-sided χ^2^ test for comparison of discrete variables and unpaired Student’s *t*-test for continuous variables.

† Selective variables were transformed into logarithmic model before unpaired Student’s *t*-test between cases and controls.

‡ Some information of concomitant asthma or family history of allergic diseases was not available in cases.

IQR, interquartile range; VAS, visual analogue scales.

### Individual SNP Association Analysis

The primary information and allele frequencies observed are listed in Table 3. All genotype distributions of control subjects were consistent with those expected from the HWE (all *P*>0.05). As shown in Table 4, the individual SNP analyses revealed that the genotype frequencies of three SNPs (SNP F1, S2, and rs2787093) were significantly different between the cases and controls (*P = *0.006 for F1, *P = *0.002 for S2, and *P* = 0.011 for rs2787093). The homozygous genotype GG of SNP F1 was associated with a significantly increased risk of mite-sensitized PER compared with the wild-type genotype CC (adjusted OR = 4.03, 95% CI = 1.49–10.93). Similar associations were also found in SNP S2 and rs2787093 (adjusted OR = 2.56, 95% CI = 1.50–4.38 for S2 and 2.24, 1.26–4.00 for rs2787093, respectively). We also found that the dominant models (MW+MM/WW) of SNP F1 (AG+GG/AA), S2 (CG+GG/CC) and rs2787093 (CT+TT/CC) showing significant associations with mite-sensitized PER (adjusted OR = 1.53, 95% CI = 1.09–2.14 for F1; 1.32, 1.02–1.70 for S2 and 1.38, 1.07–1.78 for rs2787093).

**Table 3:** Primary information of seven genotyped SNPs in the *ADAM33* gene.

SNPNo.	NCBIrs No.	Chromosomeposition*	SNP namein ref. [8]	Location	Base	MAF			*P* forHWE‡	Genotyped(%)
					change	Database†	Case	Control		
1	rs3918392	3655219	F1	exon	A>G	0.067	0.119	0.077	0.175	98.6
2	rs528557	3651742	S2	exon	C>G	0.250	0.266	0.206	0.916	98.3
3	rs2280091	3650234	T1	exon	A>G	0.078	0.093	0.072	0.782	96.6
4	rs2280090	3650205	T2	exon	G>A	0.100	0.109	0.102	0.332	98.1
5	rs3918400	3649463	V2	3′-UTR	C>T	0.159	0.188	0.163	0.300	99.1
6	rs2787094	3649161	V4	3′-UTR	C>G	0.410	0.563	0.547	0.251	99.1
7	rs2787093	3648462		3′near gene	T>C	0.233	0.282	0.226	0.145	98.0

*SNP position in NCBI dbSNP (http://www.ncbi.nlm.nih.gov/snp).

† MAF for CHB from the HapMap databases (http://www.hapmap.org) or NCBI dbSNP (http://www.ncbi.nlm.nih.gov/snp).

‡ HWE *P* value in the control group.

SNP, single nucleotide polymorphism; MAF, minor allele frequencies; HWE, Hardy-Weinberg equilibrium; CHB: Han Chinese in Beijing, China.

**Table 4:** Genotype and allele frequencies in *ADAM33* polymorphisms among cases and controls.

SNPNo.	NCBIrs No.	Genotypes	Cases		Controls		Crude OR	Adjusted OR	*P* †
			N	%	N	%	(95% CI)	(95% CI)*	
1	rs3918392		*n = *507		*n = *489				
		AA	405	79.9	419	85.7	1.00 (reference)	1.00 (reference)	**0.006**
		AG	83	16.4	65	13.3	1.32 (0.93–1.88)	1.34 (0.94–1.91)	
		GG	19	3.8	5	1.0	**3.93 (1.45–10.63)**	**4.03 (1.49–10.93)**	
		AG/GG	102	20.1	70	14.3	**1.51 (1.08–2.10)**	**1.53 (1.09–2.14)**	**0.015**
		G allele‡	0.119		0.077				**0.001**
2	rs528557		*n* = 508		*n* = 485				
		CC	288	56.7	306	63.1	1.00 (reference)	1.00 (reference)	**0.002**
		CG	170	33.5	158	32.6	1.14 (0.87–1.50)	1.15 (0.88–1.51)	
		GG	50	9.8	21	4.3	**2.53 (1.48–4.32)**	**2.56 (1.50–4.38)**	
		CG/GG	220	43.3	179	36.9	**1.31 (1.01–1.68)**	**1.32 (1.02–1.70)**	**0.040**
		G allele‡	0.266		0.206				**0.002**
3	rs2280091		*n* = 508		*n* = 468				
		AA	416	81.9	403	86.1	1.00 (reference)	1.00 (reference)	0.190
		AG	90	17.7	63	13.5	1.38 (0.98–1.96)	1.38 (0.97–1.96)	
		GG	2	3.9	2	4.3	0.97 (0.14–6.91)	0.91 (0.13–6.49)	
		AG/GG	91	17.9	64	13.7	1.38 (0.97–1.95)	1.37 (0.97–1.94)	0.073
		G allele	0.093		0.072				0.093
4	rs2280090		*n* = 505		*n* = 486				
		GG	406	80.4	394	81.1	1.00 (reference)	1.00 (reference)	0.685
		GA	88	17.4	85	17.5	1.01 (0.72–1.40)	1.00 (0.72–1.40)	
		AA	11	2.2	7	1.4	1.53 (0.59–3.97)	1.57 (0.60–4.10)	
		GA/AA	99	19.6	92	18.9	1.04 (0.76–1.43)	1.05 (0.76–1.43)	0.788
		A allele	0.109		0.102				0.609
5	rs3918400		*n* = 508		*n* = 493				
		CC	337	66.3	342	69.4	1.00 (reference)	1.00 (reference)	0.175
		CT	151	29.7	141	28.6	1.09 (0.83–1.43)	1.07 (0.81–1.40)	
		TT	20	3.9	10	2	2.03 (0.94–4.40)	2.07 (0.95–4.50)	
		CT/TT	171	33.7	151	30.6	1.15 (0.88–1.50)	1.13 (0.87–1.48)	0.305
		T allele	0.188		0.163				0.147
6	rs2787094		*n* = 508		*n* = 493				
		CC	87	17.1	95	19.3	1.00 (reference)	1.00 (reference)	0.674
		CG	270	53.2	257	52.1	1.15 (0.82–1.61)	1.14 (0.81–1.60)	
		GG	151	29.7	141	28.6	1.17 (0.81–1.69)	1.18 (0.81–1.71)	
		CG/GG	421	82.9	398	80.7	1.16 (0.84–1.59)	1.15 (0.84–1.59)	0.379
		G allele	0.563		0.547				0.462
7	rs2787093		*n* = 506		*n* = 483				
		TT	259	51.2	284	58.8	1.00 (reference)	1.00 (reference)	**0.011**
		CT	209	41.3	180	37.3	1.27 (0.98–1.65)	1.29 (0.99–1.67)	
		CC	38	7.5	19	3.9	**2.19 (1.23–3.90)**	**2.24 (1.26–4.00)**	
		CT/CC	247	48.8	199	41.2	**1.36 (1.06–1.75)**	**1.38 (1.07–1.78)**	**0.016**
		C allele‡	0.282		0.226				**0.004**

*Adjusted for age and sex in logistic regression model.

† Two-sided χ^2^ test for the distributions of genotype and allele frequencies.

‡ Assumed risk alleles.

SNP, single nucleotide polymorphism; OR, odds ratio; CI, confidence interval.

### Association and Stratification Analysis between the Individual SNP and AR-related Phenotypes

As shown in Table 5, significantly increased risk of mite-sensitized PER with asthma were found for SNP S2 (OR = 1.54, 95% CI = 1.11–2.14) and T1 (OR = 1.71, 95% CI = 1.12–2.61) under the dominant models. Comparing nonasthmatic PER patients with controls, we also found that the dominant models of F1 (OR = 1.70, 95% CI = 1.19–2.42), S2 (OR = 1.32, 95% CI = 1.01–1.74) and rs2787093 (OR = 1.39, 95% CI = 1.06–1.83) had a significantly higher risk of mite-sensitized PER than those with wild-type genotypes.

**Table 5:** Genotype frequencies of *ADAM33* polymorphisms in dominant model in PER patients with and without asthma.

SNPNo.	NCBIrs No.	Case with asthma *vs.* control		Case without asthma *vs.* control	
		Adjusted OR (95% CI)*	*P* †	Adjusted OR (95% CI)*	*P* †
1	rs3918392	1.28 (0.83–2.00)	0.258	**1.70 (1.19–2.42)**	**0.003**
2	rs528557	**1.54 (1.11–2.14)**	**0.011**	**1.32 (1.01–1.74)**	**0.048**
3	rs2280091	**1.71 (1.12–2.61)**	**0.013**	1.35 (0.93–1.95)	0.118
4	rs2280090	1.24 (0.83–1.85)	0.297	1.06 (0.75–1.49)	0.748
5	rs3918400	1.00 (0.70–1.42)	1.000	1.12 (0.84–1.50)	0.426
6	rs2787094	1.48 (0.94–2.23)	0.090	1.04 (0.74–1.47)	0.818
7	rs2787093	1.24 (0.89–1.72)	0.208	**1.39 (1.06–1.83)**	**0.018**

Dominant model: MW+MM/WW; MW: heterozygotes; MM: mutation homozygotes; WW: wild homozygotes.

*Adjusted for age and sex in logistic regression model.

† Two-sided χ^2^ test for the distributions of genotype frequencies.

PER, persistent allergic rhinitis; SNP, single nucleotide polymorphism; OR, odds ratio; CI, confidence interval.

Given the significant difference between cases and controls, the correlations between serum total IgE levels and individual locus in cases and controls were evaluated separately. No significant association was shown between total IgE levels and genotypes of each SNP in controls (all *P*>0.05). No significant difference was observed in total IgE levels among individuals with different genotypes of seven SNPs (all *P*>0.05). We also evaluated the association between other AR-related phenotypes and different genotypes of individual SNP; however, no statistical evidence was found for interactions between the different genotypes and the variables (i.e., rhinitis severity, serum specific IgE and ECP levels) (all *P*>0.05; data not shown).

### Distribution of the ADAM33 Combined Genotypes among Cases and Controls

In Table 4, the frequencies in *ADAM33* SNP F1 G (0.119 *vs.* 0.077), SNP S2 G (0.266 *vs*. 0.206), and rs2787093 C (0.282 *vs*. 0.226) alleles among the cases were significantly higher than those among the controls. Considering potential interactions of these three SNPs on the risk of mite-sensitized PER, we combined these three SNPs based on the numbers of variant (risk) alleles (SNP F1 G, S2 G and rs2787093 C).

As shown in Table 6, the combined genotypes with 0–1 variant (risk) alleles were more common (28.9% and 46.6%, respectively) and those with 2–4 variant (risk) alleles were less common (19.7%, 4.5% and 0.4%, respectively) among the controls than among the cases (23.5%, 37.4%, 25.5%, 11.5%, and 2.4%, respectively). In our study, no individual carried the combined genotypes with 5 or 6 variant (risk) alleles, presumably due to the LD. When these combined genotypes were dichotomized into two groups (i.e., zero to one *vs.* two to four risk alleles), their distributions differed significantly between the cases and controls (*P*<0.0001).

**Table 6:** Distributions of the *ADAM33* combined genotypes among cases and controls.

No. variant (risk) alleles ofthe combined alleles*	Cases (*n* = 481)		Controls (*n* = 443)		*P* †
	N	%	N	%	
0	117	23.5	135	28.9	**<0.0001**
1	186	37.4	218	46.6	
2	125	25.5	92	19.7	
3	57	11.5	21	4.5	
4	12	2.4	2	0.4	
Dichotomized groups					
0–1	303	61.0	353	75.4	**<0.0001**
2–4	194	39.0	115	24.6	

*The number represents the numbers of risk genotypes.

† Two-sided χ^2^ test for the distributions of combined genotype frequencies.

### Association and Stratification Analysis between the ADAM33 Combined Genotypes and AR-related Phenotypes

As shown in Table 7, we found that the individuals with 2–4 variant (risk) alleles had a significantly higher risk of mite-sensitized PER (adjusted OR = 1.99, 95% CI = 1.50–2.62) than those with 0–1 risk genotypes. Further stratification analysis showed that this increased risk was pronounced among subgroups of both age <18 years (adjusted OR = 1.72, 95% CI = 1.16–2.54) and age ≥18 years (adjusted OR = 2.30, 95% CI = 1.53–3.46). This increased risk was also pronounced among both male (adjusted OR = 1.87, 95% CI = 1.32–2.66) and female (adjusted OR = 2.29, 95% CI = 1.44–3.64) groups. However, no statistical evidence was observed for interactions between the combined genotypes and the variables (i.e., concomitant asthma, family history of allergic diseases, VAS score, and total IgE) (all *P*>0.05).

**Table 7:** Association and stratification analyses between the *ADAM33* combined genotypes and risk of PER.

Variables	Subcategory	N (case/control)	Combined genotypes (case/control)*				Adjusted OR(95% CI)†
			0–1		2–4		
			N	%	N	%	
Total		497/468	303/353	61.0/75.4	194/115	39.0/24.6	**1.99 (1.50–2.62)**
Age (years)							
	<18	288/230	180/171	62.5/74.4	108/59	37.5/25.7	**1.72 (1.16–2.54)**
	≥18	209/238	123/182	58.9/76.5	86/56	41.2/23.5	**2.30 (1.53–3.46)**
Sex							
	Male	329/292	207/221	62.9/75.7	122/71	37.1/24.3	**1.87 (1.32–2.66)**
	Female	168/176	96/132	57.1/75.0	72/44	42.9/25.0	**2.29 (1.44–3.64)**
Concomitant asthma							
	No	294/468	188/353	63.9/75.4	106/115	36.1/24.6	1.00 (reference)
	Yes	126/468	78/353	61.9/75.4	48/115	38.1/24.6	0.92 (0.59–1.42)
Family history of allergic diseases							
	No	293/468	188/353	64.2/75.4	105/115	35.8/24.6	1.00 (reference)
	Yes	129/468	79/353	61.2/75.4	50/115	38.8/24.6	0.87 (0.56–1.36)
VAS score							
	≤5	277/468	162/353	58.5/75.4	115/115	41.5/24.6	1.00 (reference)
	>5	220/468	141/353	64.1/75.4	79/115	35.9/24.6	1.34 (0.92–1.94)
Total IgE ‡							
	Lower level	403/468	237/353	58.8/75.4	166/115	41.2/24.6	1.00 (reference)
	Higher level	94/468	66/353	70.2/75.4	28/115	29.8/24.6	1.14 (0.72–1.82)

*The number represents the numbers of risk genotypes.

† Adjusted for age and sex in logistic regression model.

‡ Lower level: below the 90th percentile of logarithmic total IgE; Higher level: above the 90th percentile of logarithmic total IgE.

PER, persistent allergic rhinitis; VAS, visual analogue scale; OR, odds ratio; CI, confidence interval.

### Association and Stratification Analysis between the Haplotypes and Risk of AR

**Figure 1:**
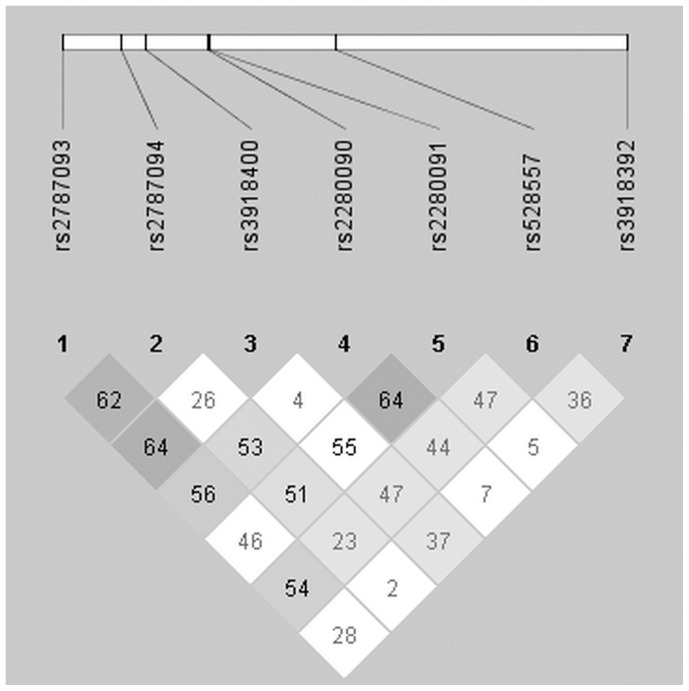
Linkage disequilibrium of seven SNPs in the *ADAM33* gene. Linkage disequilibrium (LD) of 7 SNPs was determined using the solid spine of LD option of Haploview 4.1. *D′* values are displayed in the squares. Empty red squares have a pairwise *D′* of 1.00. Red squares indicate high pairwise LD, gradually coloring down to white squares of low pairwise LD.

The LD between each pair of SNPs in *ADAM33* is presented in Fig. 1. Haplotype analysis including 7 SNPs was performed, there were >100 possible haplotypes derived from the known genotypes. Haplotypes with a frequency <0.01 in both the cases and controls were pooled into a single group, and the remaining 9 haplotypes were analyzed. As shown in Table 8, the frequency of ACAGCCT haplotype in controls was significantly higher than that in cases (*P = *0.0001, adjusted OR = 0.67, 95%CI = 0.49–0.90) compared with the common haplotype ACAGCGT, and the association was also statistically significant after Bonferroni’s correction (*P = *0.001), suggesting that ACAGCCT haplotype may have potential to protect against mite-sensitized PER. We also found AGAGCGT haplotype carrying the S2 G allele was associated with a significantly increased risk of mite-sensitized PER (adjusted OR = 2.03, 95% CI = 1.09–3.79), compared with the common haplotype ACAGCGT. However, the significance did not remain after the Bonferroni’s correction (*P = *0.256).

**Table 8:** Associations between risk of PER and frequencies of inferred haplotypes on the basis of the observed genotypes among cases and controls.

Haplotypes*	Haplotype frequencies		*P* †	Adjusted OR (95% CI)‡
	Cases	Controls		
A-C-A-G-C-G-T	0.291	0.312	0.3161	1.00 (reference)
A-C-A-G-C-C-C	0.209	0.191	0.3305	1.17 (0.90–1.52)
A-C-A-G-C-C-T	0.102	0.162	**0.0001**	**0.67 (0.49–0.90)**
A-G-A-G-T-G-T	0.054	0.059	0.6011	0.99 (0.65–1.49)
A-C-A-G-C-G-C	0.025	0.019	0.4246	1.35 (0.72–2.55)
A-G-G-A-C-G-T	0.035	0.037	0.8596	1.02 (0.62–1.70)
A-G-A-G-C-G-T	0.033	0.017	**0.0284**	**2.03 (1.09–3.79)**
A-G-A-G-C-C-T	0.027	0.024	0.6621	1.21 (0.67–2.18)
A-C-A-G-T-G-T	0.016	0.017	0.7647	0.95 (0.46–1.96)

*The alleles of haplotypes were arrayed as the location of the SNPs in *ADAM33* from 5′to 3′.

† Two-sided χ^2^ test for each haplotype *vs.* all others combined in cases and controls.

‡ Adjusted for age and sex in logistic regression model.

PER, persistent allergic rhinitis; OR, odds ratio; CI, confidence interval.

## Discussion

ADAM33 is a member of the ADAM protein family of zinc-dependent metalloproteinase superfamily [26]. We selected seven SNPs in the *ADAM33* gene and conducted an association study in a Han Chinese Han population. The results showed that three polymorphisms (SNP F1, S2 and rs2787093) were significantly associated with mite-sensitized PER and nonasthmatic mite-sensitized PER. Moreover, associations of SNPs (S2 and T1) with asthmatic mite-sensitized PER were observed in the study.

The normal structure of mucosa in nasal cavity is similar to that of bronchial mucosa, which characteristically manifests pseudostratified ciliated columnar epithelium [27]. Also, they both share the commonness of immunological process. Allergens and inflammation factors absorbed in nasal mucosa can influence lower part of airway and inflammatory infiltration of mucosa is resembled both in AR and asthma. Viewing as a whole, hyperresponsiveness present in upper airway contribute to AR and in lower airway represent asthma. ADAM33 protein has been proposed to contribute to the remodeling process present in asthma and BHR [8]. ADAM33 mRNA was seen in asthmatic subepithelial fibroblasts and smooth muscle [28] and was significantly higher in the epithelium, submucosal cells and airway smooth muscle in severe bronchial asthma [29], [30], [31]. Thus, we proposed that ADAM33 may also be implicated in the progression of PER and upper airway remodeling process.

ADAMs were originally identified as membrane-anchored proteins on the cell surface that mediate adhesion and proteolysis, and play pivotal roles in cell-to-cell interactions, cell signaling and the remodeling of extracellular matrix components [32]. ADAMs include matrix metalloproteinases (MMPs), specifically regulated by tissue inhibitors of metalloproteinases (TIMPs) during tissue remodeling. ADAM33 may release growth factors (such as EGF, TGF-β and IGF) and modify cell-surface receptor expression to stimulate the proliferation of airway mesenchymocytes in response to injury. Besides, ADAM33 might act as a dominant negative regulator, fusagen or an enhancer of a mesenchymal cell migration in the progress of airway remodeling [33], [34].

As indicated above, SNPs located in functional domain of the *ADAM33* gene may contribute to transcription and expression of ADAM33 mRNA and protein, and finally influence the function of ADAM33 in the pathogenesis of AR. For instance, F1 (Thr178Ala, in exon 6) may influence the gene expression and the composition and structure of protein. S2 is a polymorphism located in exon encoding the transmembrane domain, which is predicted to influence anchoring ADAM33 protein to the cell membrane. Although S2 encodes the synonymous exon (Gly717Gly) which does not change amino acid sequence or missense, it may alter the mRNA folding, mRNA stability and translation [35]. Further studies are warranted to reveal the influence of S2 variation to ADAM33 function. Additionally, the intracellular domain of *ADAM33* is relatively short in comparison with its nearest homologues but it is very rich in prolines and has a putative SH3 binding site (PsWPLDP) where T2 is located and may affect function [9]. T1 is in the intracellular domain that may affect bi-directional signaling. V2 and V4 located in 3′UTR and rs2787093 located in 3′near gene may affect the mRNA transcription.

Our result of the association of S2 and F1 polymorphisms in *ADAM33* with mite-sensitized PER had been previously confirmed in SAR due to Japanese cedar pollen [20]. However, the significant association of other SNPs (T1 and T2) with cedar pollinosis was observed in the Japanese patients, but not in our patients with mite-sensitized PER. The S2 polymorphism in *ADAM33* had also been reported to be associated with asthma in many case-control studies of diverse population, including Japanese [14], British [36], and Irish [13], but not American [16], Germany [37], Brazilian [38], and Colombians [24]. Several reasons can explain the variety results of association of S2 with Asthma. Firstly, it is important to consider the genetic diversity of the populations reflected in differences in the occurrence of polymorphisms and their allelic frequencies. The minor allele frequency (MAF) of S2 in Chinese may be similar to that of Japanese (MAF = 0.250 in CHB+JBT show in Table 3), yet different from that in European (MAF = 0.091 showed in the HapMap) or other populations. Secondly, environmental influence is also a crucial factor in the progression of allergic diseases. Numerous environmental exposures such as allergen, air pollution, smoking, diet, pet keeping, microbial exposure, moisture and dampness, and chemicals at the workplace have been related to the new onset of asthma [39]. Thereby, the gene effect may differ from various populations respect to their environmental exposure. Further studies should focus on the gene-environment interaction of AR and asthma to uncover the mechanism of onset and progression of the diseases.

Three SNPs (T1, T+1 and V4), which have no association with mite-sensitized PER in our study, had been previously reported to be significantly associated with AR in population of northern China [12]. The controversial results may due to its small sample size (135 cases and 151 controls) that did not have enough statistical power to reveal any significant ORs and the environmental differences between northern and eastern part of China. Also, several studies have reported no significant difference in variant genotype distributions of SNPs in the *ADAM33* gene between the asthmatics and controls [37], [40], [41], [42], [43]. More studies of larger sample size are necessary for further confirming the association between the *ADAM33* polymorphisms and AR in diverse populations.

In the analysis of the association of individual SNP with AR-related clinical phenotypes, we found that two SNPs (S2 and T1) were significantly associated with concomitant asthma in PER patients, supporting the previous findings about the relation between *ADAM33* polymorphisms and asthma. The association between *ADAM33* polymorphisms and asthma has been reported in American (T1, T2 and T+1) [8], British (S2) [15], Japanese (S2, T1 and T2) [14], and Chinese (S2, T1, T2, and V4) [12] population. Moreover, recently several meta-analysis showed that polymorphisms in the *ADAM33* gene are risk factors for asthma in the Asian (T1, V4, F+1, T2, and T+1) [44], [45] and European (S2) [46] population. However, a genome-wide association studies (GWAS) of Spanish individuals revealed that among 19 selected tag SNPs of *ADAM33*, only one SNP (rs2787095) was associated with asthma [17]. No evidence of *ADAM33* polymorphisms associated with either serum total IgE or allergen-specific IgE level was found in our study. It suggests that airway remodeling function due to activity of ADAM33 protein may not accompany with the increase of IgE production. Lack of association between *ADAM33* polymorphisms and serum total IgE levels was also reported in Japanese patients with cedar pollinosis [20] and in German and Australian Caucasian populations [19], yet was contradicted by the data from asthmatics of Dutch (S2), US white (T1 and T2) and US hispantic (T1 and T2) [16], and Colombian (T1 and T2) [24] population, suggesting an existence of race and ethnic difference.

As shown in Table 7, we found that the combined genotypes with 2–4 risk alleles were associated with a higher risk of mite-sensitized PER among subgroups of adult subjects (≥18 years old). The difference of nasal structure and development of immune system between children and adults contribute to the dissimilarity of allergic symptoms between them. Moreover, prenatal factors and early life exposures, coupled with genetically determined susceptibility from parents, have a major impact on the natural history of childhood allergy [47], [48]. According to our results, we suggested that compared with children, adults might be more susceptible to the genetic effect caused by *ADAM33*, and the effect caused by *ADAM33*, such as airway remolding. Thus, besides the discrepancy of exposure environment, pediatric allergy may differ from adult allergy in effect of genetic variation. However, lack of the analysis in the interaction of environment and genetic polymorphism may cause this finding due to chance.

In addition, we also were looking for a dose-response effect as one might expect that gene variants associated with increased airway remodeling, as suggested by Jongepier *et al.* [49], to be likewise increased in more severe AR. Actually, we did not found such relation of *ADAM33* polymorphism and rhinitis severity in patients with mite-sensitized PER. Coherent with the negative results of individual SNP with IgE, no evidence of an increased risk of PER associated with elevated serum total or specific IgE was found in combined genotypes. Nevertheless, since our sample size might not be large enough and the selected bias could not be excluded completely, the finding might be due to chance and should be interpreted with caution.

As multiple SNPs may act in combination to increase the risk of AR, we further analyzed the *ADAM33* haplotype structure and frequencies, and identified a haplotype (ACAGCCT), carrying wild-type alleles of each polymorphism, that might account for susceptibility to protect against mite-sensitized PER. Although the significance for AGAGCGT haplotype did not remain after the Bonferroni’s correction, consistent with our genotype analysis, AGAGCGT haplotype carrying S2 G allele was pronounced a borderline significantly increased risk of mite-sensitized PER.

In conclusion, our data suggested that *ADAM33* polymorphisms might play an important role in the pathogenesis of mite-sensitized PER in this Chinese population. However, functional studies are needed to elucidate the role of *ADAM33* polymorphisms in the molecular mechanisms underlying nasal allergy, and more detailed environmental exposure data are needed to confirm the effect of gene-environment interaction on mite-sensitized PER.
